# Studies of single-walled carbon nanotubes-induced hepatotoxicity by NMR-based metabonomics of rat blood plasma and liver extracts

**DOI:** 10.1186/1556-276X-8-236

**Published:** 2013-05-16

**Authors:** Bencheng Lin, Huashan Zhang, Zhiqing Lin, Yanjun Fang, Lei Tian, Honglian Yang, Jun Yan, Huanliang Liu, Wei Zhang, Zhuge Xi

**Affiliations:** 1 Institute of health and Environmental Medicine, Key Laboratory of Risk Assessment and Control Technology for Environment and Food Safety, No.1, Dali Road, Tianjin 300050, China

**Keywords:** Single-walled carbon nanotubes, Hepatotoxicity, Metabolomics, Nuclear magnetic resonance

## Abstract

The toxicological effects of single-walled carbon nanotubes (SWCNTs) were investigated after intratracheal instillation in male Wistar rats over a 15-day period using metabonomic analysis of ^1^H (nuclear magnetic resonance) NMR spectra of blood plasma and liver tissue extracts. Concurrent liver histopathology examinations and plasma clinical chemistry analyses were also performed. Significant changes were observed in clinical chemistry features, including alkaline phosphatase, total protein, and total cholesterol, and in liver pathology, suggesting that SWCNTs clearly have hepatotoxicity in the rat. ^1^H NMR spectra and pattern recognition analyses from nanomaterial-treated rats showed remarkable differences in the excretion of lactate, trimethylamine oxide, bilineurin, phosphocholine, amylaceum, and glycogen. Indications of amino acid metabolism impairment were supported by increased lactate concentrations and decreased alanine concentrations in plasma. The rise in plasma and liver tissue extract concentrations of choline and phosphocholine, together with decreased lipids and lipoproteins, after SWCNTs treatment indicated a disruption of membrane fluidity caused by lipid peroxidation. Energy, amino acid, and fat metabolism appeared to be affected by SWCNTs exposure. Clinical chemistry and metabonomic approaches clearly indicated liver injury, which might have been associated with an indirect mechanism involving nanomaterial-induced oxidative stress.

## Background

Carbon nanotubes are allotropes of carbon with a cylindrical nanostructure and categorized as single-walled (SWCNTs) and multi-walled nanotubes. By virtue of their unique properties, SWCNTs have been demonstrated as promising nanomaterials for a wide range of applications. In particular, increasing attention has been directed to their utilization in biomedicine, such as in biosensors, drug delivery, and biomarkers [1,2]. However, attention has also been directed toward human health effects that exposure to these materials may produce. Thus, nanotoxicology has become an important research topic in nanoscience.

In the past decade, various groups have independently reported toxicological studies on SWCNTs, both *in vitro* and *in vivo*. These results have mainly focused on pulmonary toxicity, cytotoxic effects, inflammatory response, and genotoxicity [3-9]. However, the studies on SWCNTs leading to hepatotoxicity in animals have been limited in scope [10,11], and they only assessed the effects of SWCNTs on reactive oxygen species induction and various hepatotoxicity markers (alanine aminotransferase (ALT), aspartate aminotransferase (AST), alkaline phosphatase (ALP), LPO, and liver morphology) in the mouse model.

Recent studies have shown that metabonomic methods are useful in the assessment of toxic mechanisms and prediction of toxicity [12,13]. Nuclear magnetic resonance (NMR) spectroscopy is one of the major techniques used in metabonomic studies as these spectra can contain a wealth of metabolic information. The signals from thousands of individual metabolites can be observed simultaneously and can partially overlap [14]. Processing these complex data can be simplified by multivariate statistical analysis, including data reduction and pattern recognition techniques, such as principal components analysis (PCA) and partial least squares discriminant analysis [15]. Metabolic changes induced by the administration of a xenobiotic can be visualized clearly by NMR spectroscopy and pattern recognition techniques [16,17].

Herein, the hepatotoxicity in rats exposed to SWCNTs by intratracheal instillation was explored using a ^1^H NMR-based metabonomic approach to examine blood plasma and liver tissue extracts obtained from rats treated with different SWCNTs concentrations. Concurrently, the toxic threshold and identification of potentially useful toxicity biomarkers of SWCNTs-induced hepatotoxicity were also studied by conventional clinical chemistry and histopathological examinations.

## Methods

### Single-walled carbon nanotubes and suspension preparation

**Figure 1:**
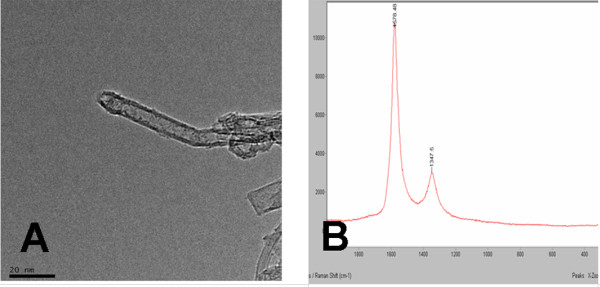
**The non-functionalized SWCNTs.** (**A**) TEM of SWCNTs. (**B**) Raman spectra of SWCNTs.

Non-functionalized SWCNTs, produced by CoMoCAT® (Sigma-Aldrich, St. Louis, MO, USA) catalytic CVD process, were purchased from Sigma-Aldrich, Inc. (St. Louis, MO, USA). Their diameter of 0.8 to 1.2 nm and a length of several microns were determined by transmission electron microscopy (TEM, JEM-2010FEF, JEOL, Ltd., Tokyo, Japan) (Figure 1A). Raman spectroscopy had been used to assess purity (Raman spectrometer, RM200, Renishaw, Gloucestershire, UK) (Figure 1B). The carbon content and the proportion of carbon as SWNT were above 90% and 70%, respectively.

SWCNTs samples at 150, 300, and 450 mg were dispersed in 20-mL volumes of 0.9% sodium chloride solution, followed by ultrasonication at <50°C for 5 h. The resulting SWCNTs concentrations were 7.5, 15, and 22.5 mg/mL, respectively.

### Ethics statement

All experiments involving the care and use of animals were performed in accordance with the guidelines and regulations concerning the ethics of science research in the Institute of Health and Environmental Medicine and approved by the Ethics Review Board of the Institute of Health and Environmental Medicine (approval number JKYSS-2009-018).

### Animals and treatment

Thirty healthy male Wistar rats (8 weeks of age, weight 180 to 220 g) were obtained from the Academy of Military Medical Sciences (Beijing, China). All procedures concerning animal usage were reviewed and approved by the Institutional Animal Care and Use Committee of the Academia. All rats were housed individually in metabolic cages and, throughout the study period, allowed food and tap water *ad libitum*, with light/dark cycles altering every 12 h, environment at 18°C to 22°C, and humidity from 40% to 60%. After 1 week of acclimatization, weight-matched rats were divided randomly into four groups (*n* = 6 per group) comprising a sodium chloride group (control) and low-, medium-, and high-concentration groups (7.5, 15, and 22.5 mg/kg body weight and named SWCNTs-L, M, and H, respectively). The rats were exposed to SWCNTs by intratracheal instillation of the corresponding SWCNTs suspensions once a day for 15 consecutive days, with the control group treated concurrently with 0.9% sodium chloride solution.

### Detection of biochemistry parameters

Blood samples were collected from the rats' eyes 24 h after the final exposure. Samples were allowed to clot at 4°C for 60 min and centrifuged at 3,500 × *g* at 4°C for 10 min to remove precipitates. Then, plasma biochemistry parameters, including ALT, AST, ALP, albumin (ALB), total protein (TP), and total cholesterol (TC), were analyzed using a Hitachi 7020 automatic analyzer (Hitachi, Tokyo, Japan).

### Histopathological evaluation

After the rats were euthanized, the left lateral lobes of each liver were embedded in paraffin and thin sectioned coronally. The sections were then stained with hematoxylin-eosin for examination by light microscopy.

### ^1^H NMR spectroscopic measurement of blood plasma

Sample preparation and NMR analyses were conducted as previously described [18,19]. Briefly, 400 μL of plasma was mixed with 200 μL of D_2_O and 100 μL of a 1-mg/mL solution of trimethylsilyl propanoate in D_2_O and then transferred to 5-mm NMR tubes. Samples were analyzed by ^1^H NMR spectroscopy using a Varian INOVA-600 spectrometer (Varian Medical Systems, Inc., Palo Alto, CA, USA). Two types of ^1^H NMR spectra were acquired for each sample, with water-suppressed Carr-Purcell-Meiboom-Gill (CPMG) spectra acquired using a pulse sequence acting as a T_2_ relaxation filter to suppress signals from macromolecular motion and other molecules with constrained molecular motions. Water-suppressed diffusion-edited spectra were acquired to remove peaks from low molecular weight components using a bipolar-pair longitudinal decay current (LED) pulse sequence.

### ^1^H NMR spectroscopic measurement of aqueous soluble liver extracts and lipid-soluble liver extracts

Liver tissue extracts were prepared based on a procedure reported [20,21]. Here, 250-mg samples of frozen liver tissue were homogenized with 2 mL of 50% acetonitrile in an ice/water bath. After standing in ice for 10 min, the extraction samples were centrifuged at 5,100 rpm and 4°C for 15 min, and the aqueous layer and precipitates were recovered.

The aqueous layer was removed and lyophilized before precipitate removal by resuspension in 600 μL of sodium phosphate buffer in D_2_O (0.1 M, pH 7.4), containing 60 μL of 0.1% sodium TSP, and centrifugation at 14,000 rpm at 4°C for 8 min. The resulting solutions were transferred to 5-mm NMR tubes, and NMR spectrum was acquired with water signals suppressed by presaturation, as described above. Sixty-four free induction decays (FIDs) were collected into 64K data points over a spectral width of 9,000 Hz with 2-s relaxation delay and acquisition time. The FIDs were weighted using an exponential function with a 0.5-Hz line-broadening factor prior to Fourier transformation.

The precipitates were collected into polypropylene tubes containing 2-mL solution of 75% chloroform and 25% methanol. The extraction was followed by a further centrifugation (5,000 × *g* for 15 min). The lipophilic supernatants were removed, then dried under a stream of nitrogen. After adding 600 μL of deuterated chloroform, the samples were recentrifuged at 14,000 rpm for 8 min, then the lower phases were transferred to 5-mm NMR tubes, and NMR spectra were acquired.

### Data reduction and pattern recognition analysis of ^1^H NMR spectra

All NMR spectra were phased and baseline corrected, and then, the data were reduced to 225 integrated regions of equal width (0.04 ppm) corresponding to the region from δ9.38 to δ0.22 using the VNMR 6.1C software package (Varian, Inc.). Each data point was normalized to the sum of its row (i.e., to the total integral for each NMR spectrum) to compensate for variations, and the values of all variable means were centered and Pareto scaled before PCA was applied using the SIMCA-P software package (v10, Umetrics AB, Umea, Sweden). Pareto scaling provided each variable a variance numerically equal to its standard deviation. Score plots of the first two principal components (PCs) were used to visualize group separations, and the PC loading values reflected the NMR spectra regions that were altered as a result of nanotube exposure [14,17].

### Statistical analyses

Data were presented as mean ± standard deviations. Statistical analyses were performed using SPSS software, version 13.0 (SPSS Inc., Chicago, IL, USA). A one-way analysis of variance and Bartlett's test were calculated for each sampling value. A *p* value less than 0.05 was regarded as statistically significant.

## Results

### Effects of SWCNTs on biochemical indicators of rat liver function

After intratracheal instillation for 15 days, rat plasma AST, ALB, ALT, ALP, TP, and TC values were measured as indicators of liver function. Compared with the control group, the ALP, TP, and TC concentrations in the SWCNTs-H group decreased significantly (*p* < 0.05). Also, the ALB and TP concentrations in the SWCNTs-H group decreased compared with the SWCNTs-L group (*p* < 0.05, Table 1).

**Table 1:** Effects of SWCNTs on biochemical indicators of rat liver function

**Group**	**AST (g/L)**	**ALB (g/L)**	**ALT (g/L)**	**ALP (g/L)**	**TP (g/L)**	**TC (μmol/L)**
Control	156.9 ± 39.0	49.8 ± 14.9	49.0 ± 9.4	427.2 ± 57.9	82.2 ± 5.4	1.95 ± 0.34
SWCNTs-L	125.1 ± 16.7^a^	42.0 ± 1.3	50.8 ± 5.4	374.5 ± 81.5	78.3 ± 2.6	1.68 ± 0.15
SWCNTs-M	127.6 ± 12.5	39.9 ± 1.4	53.7 ± 9.1	345.5 ± 90.1	75.9 ± 1.4^a^	1.83 ± 0.14
SWCNTs-H	129.9 ± 18.9	39.2 ± 1.5^b^	51.2 ± 9.6	317.8 ± 41.2^a^	71.8 ± 4.4^a,b^	1.59 ± 0.18^a^

AST, aspartate aminotransferase; ALB, albumin; ALT, alanine aminotransferase; ALP, alkaline phosphatase; TP, total protein; TC, total cholesterol. ^a^Compared with the control group, *p* < 0.05. ^b^Compared with the SWCNTs-L group, *p* < 0.05.

### Histopathological evaluation

**Figure 2:**
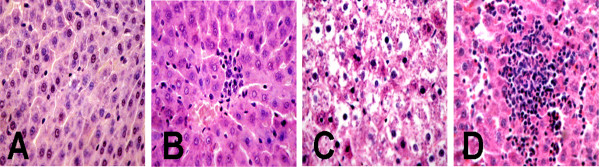
**Light micrographs of liver tissue of rats exposed to SWCNTs.** (**A**) Control group liver and (**B**, **C**, **D**) SWCNTs-L, SWCNTs-M, and SWCNTs-H group livers, respectively. Magnification, ×200.

The histological changes of the livers in the control group after treatment revealed no observable damage (Figure 2A). In contrast, the livers of the experimental groups (Figure 2B,C,D) produced mild to moderate cellular swelling in the liver centrilobular portion, focal necrosis, and inflammatory cell infiltration, especially in the SWCNTs-M and SWCNTs-H groups (Figure 2C,D), indicating that SWCNTs caused liver damage under these conditions.

### ^1^H NMR spectroscopic and pattern recognition analysis of rat plasma

**Figure 3:**
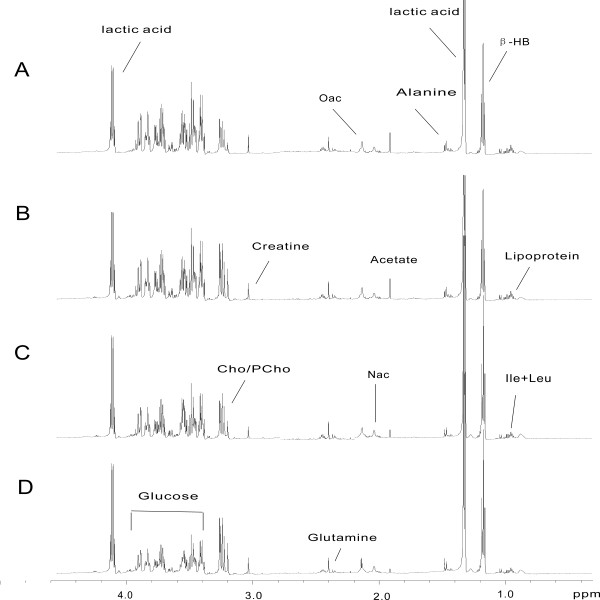
^**1**^**H NMR spectra of plasma samples (CPMG) after exposed to SWCNTs in rats.** (**A**) Control group and (**B**, **C**, **D**) SWCNTs-L, SWCNTs-M, and SWCNTs-H groups, respectively.

**Figure 4:**
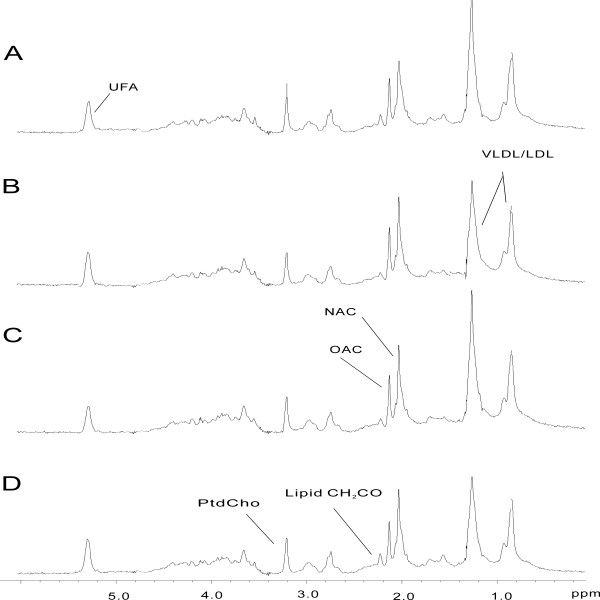
^**1**^**H NMR spectra of plasma samples (LED) after exposed to SWCNTs in rats.** (**A**) Control group and (**B**, **C**, **D**) SWCNTs-L, SWCNTs-M, and SWCNTs-H groups, respectively.

^1^H NMR spectra of plasma included spin-echo and diffusion-edited NMR spectra, which reflected the lower molecular weight and macromolecular weight metabolites, respectively, present in the plasma. In the analysis of the ^1^H NMR spectra, the intensities of some endogenous metabolite signals changed as a consequence of SWCNTs administration (Figures 3 and 4). These changes were evident as relative increases in lactic acid and choline concentrations and decreases in the concentrations of alanine, blood sugar, blood fat, and low-density lipoprotein (LDL), compared to control values.

**Figure 5:**
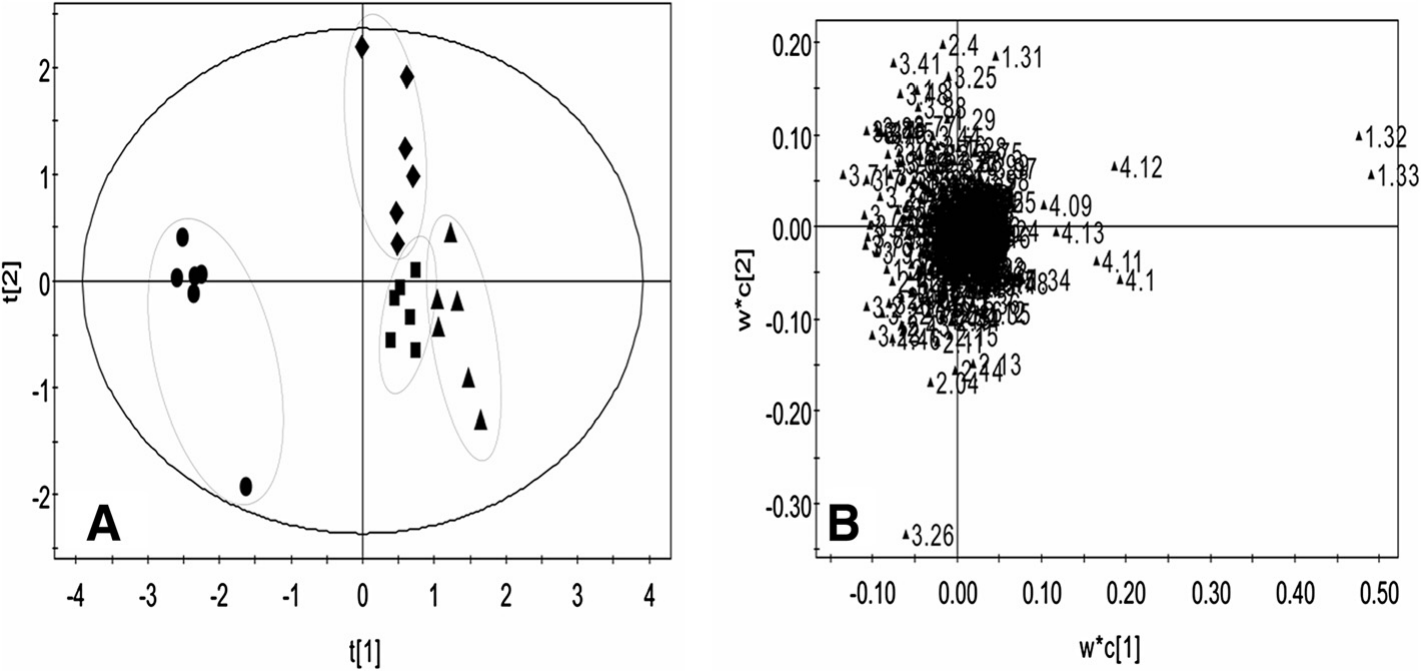
**CPMG score plot (A) and loading plot (B) for the endogenous metabolite profiles in plasma samples after exposed to SWCNTs in rats.** Control (diamond), SWCNTs-L (square), SWCNTs-M (triangle), and SWCNTs-H (circle) groups. In the score plot, each data point represents one rat sample, and the distance between points in the score plot is an indication of the similarity between samples. In the loading plot, each data point represents one bucket. The plot identifies which spectral regions are responsible for the differences between the spectra observed in the score plot.

In score plot of PCA, each data point represents one rat sample, and the distance between points in the score plot is an indication of the similarity between samples. In loading plot for the corresponding score plot, each data point represents one bucket (with the chemical shift indicated explicitly). The plot identifies which spectral regions (and thus which chemical compounds) are responsible for the differences between the spectra observed in the score plot. The PCA score plot derived from the ^1^H NMR plasma spectra of low molecular weight metabolites showed that control and dosed groups were well separated on the plot (Figure 5A). The loading plot showed that lactate (δ1.31-1.33, 4.10-4.12), glucose (δ3.46), glutamine (δ2.42-2.44), lipoprotein (δ0.9, 1.7), alanine (δ1.48), and creatine (δ3.03) were among the components that contributed markedly to the separation of the groups (Figure 5B).

**Figure 6:**
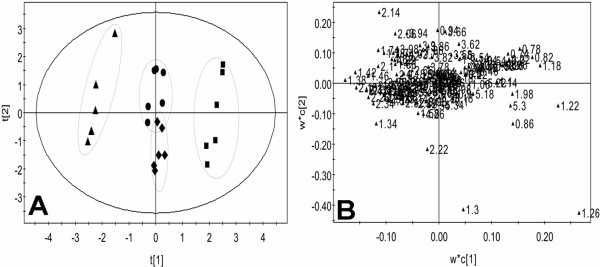
**LED score plot (A) and loading plot (B) for the endogenous metabolite profiles in plasma samples after exposed to SWCNTs in rats.** Control group (diamond), SWCNTs-L (square), SWCNTs-M (triangle), and SWCNTs-H (circle) groups.

The PCA score plot (Figure 6A) from the ^1^H NMR plasma spectra of macromolecular weight metabolites showed that the SWCNTs-L and SWCNTs-M groups were separated from the control group, but the observed overlapping in the score plot of the SWCNTs-H and control groups indicated that their differences were not obvious. As Figure 6B shows, most points were located around the origin point, and only a few points were away from the origin. The significant differences between each group were caused by the compound represented by these scattered points. Inspection of the loading SWCNTs suggested that the metabolic effects following SWCNTs treatments were characterized by significant changes in very low density lipoprotein (VLDL) and LDL, (δ0.82, δ0.86, δ1.26) and phosphatidylcholine (δ3.22) as well as several unknown materials (δ1.22, δ1.3), which require further study (Figure 6B). The SWCNTs-induced variations in plasma endogenous metabolites are summarized in Table 2.

**Table 2:** Summary of rat plasma metabolite variations induced by SWCNTs administration

**Chemical shift (δ, ppm)**	**Metabolites**	**SWCNTs-L group**	**SWCNTs-M group**	**SWCNTs-H group**
0.80-0.90, 1.20-1.29	Lipoprotein	↓	↓	↑
0.94	Ile + Leu	↑	↑	↑
1.31-1.33, 4.10-4.12	Lactate	↑	↑	↑
1.48	Alanine	↓	↓	↓
1.91	Acetate	↓	↓	↑
2.03-2.04	NAc	↑	↑	↑
2.13-2.14	OAc	↑	↑	↑
2.42-2.44	Gln-glutamine	↑	↑	↑
3.03	Creatine	↓	↓	↑
3.20	Cho	↑	↑	↑
3.22, 3.23	PCho	↑	↑	↑
3.40-4.00	Glucose	↓	↓	↓
0.70	HDL	↑	↓	↑
0.82, 0.86	VLDL/LDL	↓	↓	↓
1.10	HDL	↑	↓	↑
1.26	VLDL/LDL	↓	↓	↓
1.58	Lipid CH_2_CH_2_CO	↓	↑	↓
2.02	NAc	↑	↓	↑
2.14	OAc	↓	↑	↑
2.26	Lipid CH_2_CO	↓	↑	↓
3.22	PtdCho	↓	↑	↓
5.30	UFA	↑	↓	↑

Ile, isoleucine; Leu, leucine; NAc, *n*-acetylgalactosamine; OAc, *O*-acetyl glucoprotein; Cho, choline; PCho, phosphatidylcholine; HDL, high-density lipoprotein; VLDL, very low density lipoprotein; LDL, low-density lipoprotein; PtdCho, phosphatidylcholine; UFA, unesterified fatty acids. Down arrow indicates decrease, and up arrow indicates increase, compared to control.

### ^1^H NMR spectroscopic and pattern recognition analysis of aqueous soluble liver extract

**Figure 7:**
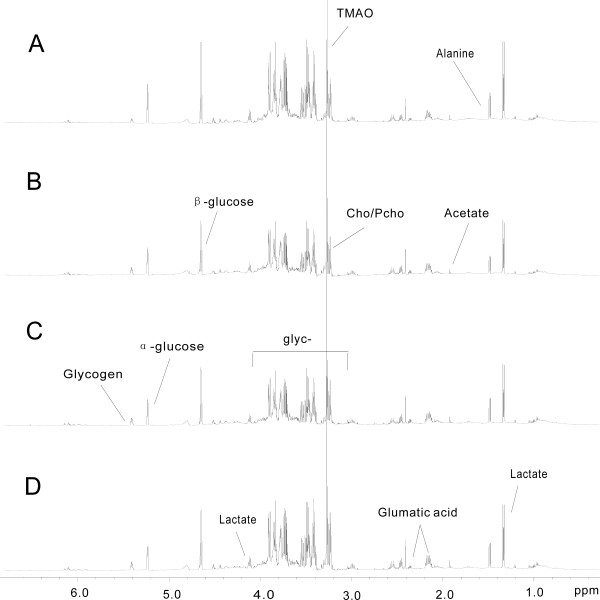
^**1**^**H NMR spectra of rat aqueous soluble liver tissue extracts after exposed to SWCNTs in rats.** (**A**) Control group and (**B**, **C**, **D**) SWCNTs-L, SWCNTs-M, and SWCNTs-H groups, respectively.

**Figure 8:**
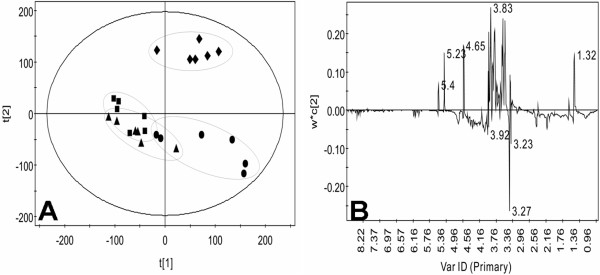
**Score (A) and loading (B) plots for the endogenous metabolite profiles in aqueous soluble liver extracts after exposed to SWCNTs in rats.** Control (diamond), SWCNTs-L (square), SWCNTs-M (triangle), and SWCNTs-H (circle) groups.

Typical ^1^H NMR spectra of aqueous soluble liver extract following administration of SWCNTs are shown in Figure 7. Examination of the score plot (Figure 8A) from ^1^H NMR spectra of samples from the control and dosed groups indicated that the control group was separated from the three treated groups, but the three treated groups overlapped with each other. It revealed that SWCNTs could cause cell oxidative damage, but the dose-related hepatotoxicity was not obvious.

Examination of the PCA loading plot (Figure 8B) in combination with the subsequent inspection of the corresponding ^1^H NMR spectra showed that lactate (δ1.32-1.34, 4.09-4.12), alanine (δ1.47-1.49), trimethylamine oxide (δ3.27), choline, phosphocholine (3.22, 3.23), β-amylaceum (δ4.65), α-amylaceum (δ5.32), and glycogen (δ5.40, 5.41), as well as several unknown materials (δ3.83, δ3.92), which require further study, were among the components that contributed markedly to the separation of the groups. The dominant metabolites in aqueous soluble liver extracts that influenced the differentiation between the control and treatment samples are summarized in Table 3.

**Table 3:** Summary of metabolite variations induced by SWCNTs in rat aqueous soluble liver tissue extract

**Chemical shift (δ, ppm)**	**Metabolites**	**SWCNTs-L group**	**SWCNTs-M group**	**SWCNTs-H group**
1.32-1.34, 4.09-4.12	Lactate	↓	↓	↓
1.47-1.49	Alanine	↓	↓	↓
2.04-2.06, 2.13, 2.14, 2.36	Glutamate	↑	↑	↑
3.22, 3.23	Cho/PCho	↑	↑	↑
3.27	TMAO	↑	↑	↑
3-4	glyc-	↓	↓	↓
4.65	β-glucose	↓	↓	↓
5.23	α-glucose	↓	↓	↓
5.40, 5.41	Glycogen	↓	↓	↓

Cho, choline; PCho, phosphatidylcholine; TMAO, trimethylamine oxide. Down arrow indicates decrease, and up arrow indicates increase, compared to control.

### ^1^H NMR spectroscopic and pattern recognition analysis of lipid-soluble liver extracts

**Figure 9:**
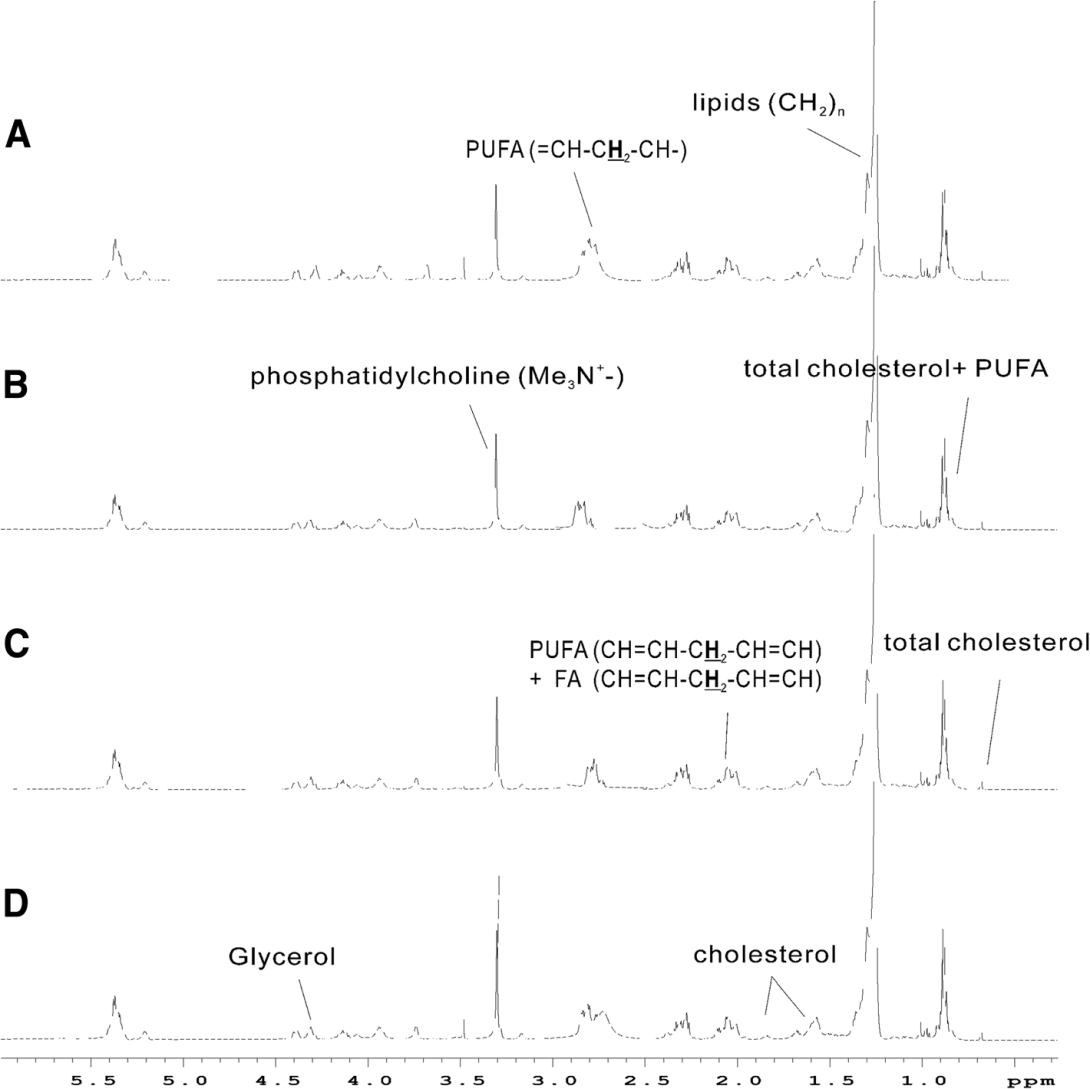
^**1**^**H NMR spectra of rat lipid-soluble liver extracts after exposed to SWCNTs in rats.** (**A**) Control group and (**B**, **C**, **D**) SWCNTs-L, SWCNTs-M, and SWCNTs-H groups, respectively.

**Figure 10:**
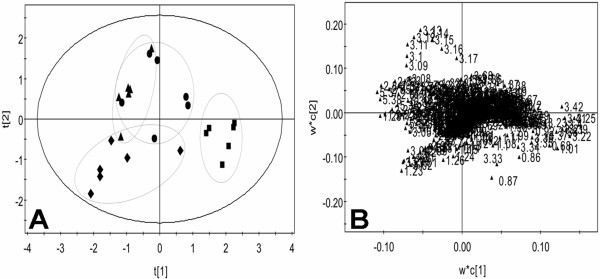
**Score (A) and loading (B) plots for the endogenous metabolite profiles in lipid-soluble liver extracts after exposed to SWCNTs in rats.** Control (diamond), SWCNTs-L (square), SWCNTs-M (triangle), and SWCNTs-H (circle) groups.

Typical ^1^H NMR spectra of lipid-soluble liver extracts following administration of SWCNTs are shown in Figure 9. Comparison of the ^1^H NMR spectra of samples from the control and dosed groups indicated that the medium and high groups overlapped on the score plot (Figure 10A), but the differences between the control and low groups were obvious.

Examination of the PCA loading plot (Figure 10B) in combination with the subsequent inspection of the corresponding ^1^H NMR spectra showed that polyunsaturated fatty acid (δ0.89, 2.00, 2.76), lipids (δ1.26, 1.58), and cholesterol (δ1.05-1.18, 1.51) were among the components that contributed markedly to the separation of the groups (Figure 9). The dominant metabolites influencing the differentiation between control and treatment samples are summarized in Table 4.

**Table 4:** Summary of metabolite variations induced by SWCNTs in lipid-soluble rat liver tissue extract

**Chemical shift (δ, ppm)**	**Metabolites**	**SWCNTs-L group**	**SWCNTs-M group**	**SWCNTs-H group**
0.66	Total cholesterol	↑	↓	↓
0.89	Total cholesterol + PUFA (CH_3_)	↓	↑	↓
1.05-1.18	Cholesterol	↑	↓	↓
1.26	Lipids (-CH_2_-CH_2_-CH_2_-)	↓	↓	↓
1.51	Cholesterol	↑	↑	↑
1.58	Lipids (CH_2_CH_2_CO)	↓/-	↑/-	↓/-
1.82	Cholesterol	↑	↑	↑
2.00	PUFA (CH=CH-CH_2_-CH=CH) FA (CH=CH-CH_2_-CH=CH)	↓	↓/-	↓
2.76	PUFA (=CH-CH_2_-CH-)	↓	↑	↓
3.30	Phosphatidylcholine (Me^3^N^+-^)	↓	↓	↑
4.27	Glycerol in PC/PE/TG	↑	-	↑

PUFA, polyunsaturated fatty acid; FA, fatty acid. Down arrow indicates decrease; up arrow indicates increase; and a hyphen means no change, compared to control.

## Discussion

Recent studies have shown that metabonomic approach can be used as a rapid analytical tool for the study on effects of hepatotoxic compounds [22-24]. In this study, NMR-based metabonomic methods coupled with traditional clinical chemistry and histopathology methods were used to demonstrate SWCNTs exposure-induced hepatotoxicity in rats. The complex disturbances in the endogenous metabolite profiles of rat biofluids combined with remarkable histopathological evidence and the change of the plasma enzyme concentrations could be related to nanoparticle-induced hepatotoxicity.

SWCNTs were found here to show effects on the chemistry and histopathology of rat blood and liver. Obviously, changes were observed in clinical chemistry features, including ALP, TP, and TC, and in liver pathology (Table 1 and Figure 2, respectively), suggesting that SWCNTs clearly have hepatotoxic abilities in rats. The release of cellular hepatospecific enzymes, such as ALP, might have resulted from nanoparticle-induced damage of cell membrane integrity, and the observed reduced TP suggested perturbation of protein biosynthesis and catabolism. From these observations, SWCNTs appeared to produce hepatotoxicity via discrete pathophysiologic necrosis and inflammation. The obtained PCA data were in good agreement with the histopathology and clinical chemistry data, with the metabonomic analytical results being more sensitive than clinical chemistry analyses.

The PCA of ^1^H NMR data showed that, in rat plasma and liver tissue, SWCNTs exposure altered the concentrations of glutamate, creatine, lactate, TMAO, cho, HDL, VLDL, and glucose and that these altered metabolites might be considered possible biomarkers for such hepatotoxicity. SWCNTs exposure appeared to induce energy metabolism disturbances, with choline and phosphocholine being breakdown products of phosphatidylcholine, the major membrane constituent. After SWCNTs treatment, the observed rise in plasma choline and phosphocholine concentrations, together with a drop in plasma lipids and lipoproteins, denoted a disruption of membrane fluidity caused by lipid peroxidation [25].

The increased glutamine concentration in aqueous soluble extracts of liver tissues resulted from the cytosolic accumulation of glutamine, which was due to defective GSH transport from the cytosol into the mitochondria, as a result of decreased membrane fluidity due to the decreased content of unsaturated fatty acids in cellular membranes [14,26]. The glucose concentrations in plasma spectra and those of glucose and glycogen in aqueous soluble liver extract were decreased significantly in rats after SWCNTs treatment, which suggested that the rates of glycogenolysis and glycolysis increased because of inhibited lipid metabolism in these animals.

SWCNTs exposure appeared to induce perturbations in amino acid metabolism. Alanine and glucose concentrations are associated with the glucose-alanine cycle [14]. The change of alanine and glucose concentrations in plasma and aqueous liver tissue extracts from SWCNTs-treated rats implied nanoparticle-induced perturbations of the glucose-alanine cycle.

## Conclusions

The present investigation demonstrated that exposure to SWCNTs induced significant hepatotoxicity in rats. The results suggested that SWCNTs inhibited mitochondrial function by altering energy and lipid metabolism, which resulted in free fatty acid and lactate accumulation. The NMR-based metabonomic approach applied here represents a promising and sensitive technique for examining SWCNTs toxicity in an animal model. Further studies are necessary to verify these metabolites as useful biomarkers for SWCNTs hepatotoxicity assessment.

## Competing interests

The authors declare that they have no competing interests.

## Authors' contributions

BCL and HSZ participated in the design of the study, carried out the experiments, and drafted the manuscript. ZQL and YJF modified the draft of the manuscript. LT, HLY, and HLL performed the statistical analysis. JY and WZ checked the manuscript grammar. ZGX designed the study and guided this work. All authors read and approved the final manuscript.
